# Review: Precise sarcoma patient-derived orthotopic xenograft (PDOX) mouse models enable identification of novel effective combination therapies with the cyclin-dependent kinase inhibitor palbociclib: A strategy for clinical application

**DOI:** 10.3389/fonc.2022.957844

**Published:** 2022-08-08

**Authors:** Takashi Higuchi, Kentaro Igarashi, Norio Yamamoto, Katsuhiro Hayashi, Hiroaki Kimura, Shinji Miwa, Michael Bouvet, Hiroyuki Tsuchiya, Robert M. Hoffman

**Affiliations:** ^1^ AntiCancer, Inc., San Diego, CA, United States; ^2^ Department of Surgery, University of California San Diego, San Diego, CA, United States; ^3^ Department of Orthopaedic Surgery, Graduate School of Medical Sciences, Kanazawa University, Kanazawa, Japan

**Keywords:** patient-derived orthotopic xenograft (PDOX), sarcoma, CDK4/6 inhibitor, palbociblib, osteosarcoma, soft-tissue sarcoma, combination therapy, methioninase

## Abstract

**Introduction:**

Sarcomas are rare heterogeneous malignant tumors that originate and develop in soft tissue or bone. Effective treatment for sarcomas is still limited to traditional chemotherapy and surgery that are often ineffective for recurrent disease. Cyclin-dependent kinases (CDKs) promote abnormal cell cycling and cell division in many cancers including sarcomas. Therefore, our hypothesis was that CDK inhibitors may be useful candidates for sarcoma treatment. Patient-derived orthotopic xenograft (PDOX) mouse models mimic the clinical disease for all major cancer types and have identified effective treatments that hold much clinical promise. The present report reviews sarcoma PDOX models that we have established for their potential to discover effective combination treatments based on CDK inhibitors for recalcitrant sarcoma.

**Methods:**

We have previously reported six sarcoma PDOX studies evaluating the CDK inhibitor palbociclib on sarcoma, including osteosarcoma, Ewing sarcoma, de-differentiated liposarcoma, and peritoneal metastatic leiomyosarcoma.

**Results:**

Palbociclib monotherapy significantly inhibited, but not regressed, the PDOX growth of osteosarcoma, Ewing sarcoma, de-differentiated liposarcoma, and peritoneal metastatic leiomyosarcoma. A combination of palbociclib and a mammalian target of rapamycin (mTOR) inhibitor, everolimus, significantly inhibited, but did not regress, the PDOX growth of osteosarcoma. Combinations of palbociclib with a multikinase inhibitor, sorafenib, and palbociclib combined with recombinant methioninase were effective and regressed the osteosarcoma and de-differentiated liposarcoma PDOX models, respectively.

**Conclusions:**

Novel effective drug combinations using the CDK inhibitor palbociclib were identified in PDOX models of the major types of sarcomas. Methionine restriction effected by methioninase increased the efficacy of palbociclib. Combination therapy with palbociclib is a promising future strategy for improved sarcoma therapy in the clinic.

## Introduction

Sarcomas are rare heterogeneous malignant tumors that originate and develop in connective tissue, such as muscle, adipose, vascular, nerve, and bone (1). Their rarity and heterogeneous features have limited the development of more effective therapies (2). Cyclin-dependent kinases (CDKs) regulate cell cycle progression and abnormal activation, or overexpression of CDKs results in altered cell cycle behavior in many cancers (3, 4). Recent studies suggest that CDKs are major drivers of sarcomagenesis (5). Therefore, our hypothesis was that CDK inhibitors may be useful candidates for sarcoma treatment.

Our laboratory developed the patient-derived orthotopic xenograft (PDOX) mouse model for all major cancer types (6). The PDOX models mimic the clinical disease, including the malignant behavior of sarcoma, due to their natural tumor microenvironment (7–11). The PDOX models, established from patients with many cancer types, including sarcoma, have identified effective treatments that hold much clinical promise (1, 12). The present report reviews sarcoma PDOX models thus far established and their potential to discover effective combination treatment, based on CDK inhibitors, for recalcitrant sarcoma.

## The cyclin-dependent kinase 4/6 inhibitor, palbociclib, inhibits a cyclin-dependent kinase inhibitor 2A-deletion Ewing sarcoma in a patient-derived orthotopic xenograft model

The efficacy of a CDK inhibitor, palbociclib, was evaluated in a PDOX model established from a chest-wall Ewing sarcoma patient who had undergone conventional chemotherapy including doxorubicin, vincristine, and cyclophosphamide (13). Ewing sarcoma is the second most common sarcoma of bone in children and young adults comprising poorly differentiated small round cells (13, 14).

Palbociclib is the first selective CDK4/6 inhibitor approved for cancer treatment and is used for estrogen receptor-positive/human epidermal growth factor receptor-2 (HER2)-negative metastatic breast cancer (15). Since deletion of cyclin-dependent kinase inhibitor 2A (CDKN2A), which is a tumor-suppressor gene and a negative regulator of CDK4/6, was found in this Ewing sarcoma patient's tumor, the efficacy of palbociclib was tested on the PDOX model of this tumor (13).

**Figure 1:**
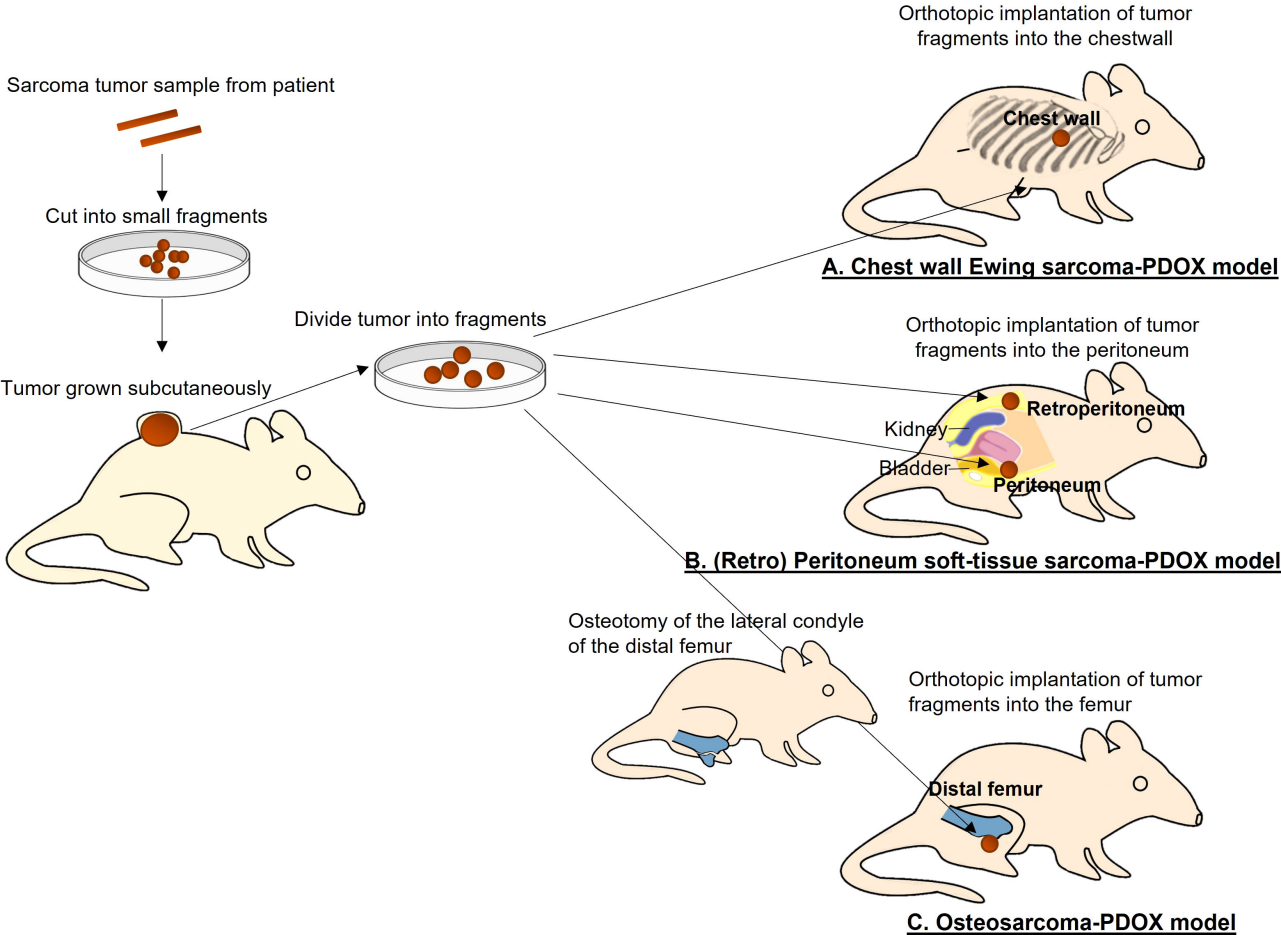
Establishment of a patient-derived orthotopic xenograft (PDOX) models of sarcoma. **(A)** Chest-wall Ewing sarcoma PDOX model. **(B)** Peritoneum and retroperitoneum soft-tissue sarcoma PDOX models. **(C)** Osteosarcoma PDOX model.

The Ewing sarcoma PDOX model was established using the surgical orthotopic implantation (SOI) technique that we developed (6), implanting a single tumor fragment orthotopically into the right chest wall of nude mice (**Figure 1A**) (13). Histological features of the PDOX tumor were similar to those of the original patient, demonstrating small round cells. Palbociclib (100 mg/kg) was orally administered to the Ewing sarcoma PDOX models for 21 consecutive days. Palbociclib significantly inhibited PDOX tumor growth, while first-line treatment doxorubicin did not affect the growth of PDOX. It has been reported that 10%–22% of Ewing sarcoma and 20% of all sarcoma types have a CDKN2A deletion (16–18). This study demonstrated that palbociclib appears to be a clinically promising agent for Ewing sarcoma and possibly other sarcomas that have a CDKN2A deletion. Three clinical trials investigating the efficacy of palbociclib for Ewing sarcoma are ongoing (ClinicalTrials.gov).

## Palbociclib in soft-tissue sarcoma patient-derived orthotopic xenograft models

The efficacy of palbociclib was next evaluated on recalcitrant soft-tissue sarcoma PDOX models. Liposarcoma and leiomyosarcoma are two of the most common subtypes of soft-tissue sarcoma. Almost half of high-grade soft-tissue sarcoma patients develop local or distal recurrence and their prognosis is poor, with a median overall survival of 20 months (19, 20).

Leiomyosarcoma, which frequently occurs in the extremities, the retroperitoneal space, and uterus, has a high risk of metastasis and local recurrence with a 5-year recurrence rate of 40%, leading to high mortality (21). Doxorubicin as first-line treatment and ifosfamide, gemcitabine and docetaxel, eribulin, pazopanib, and trabectedin as second-line treatment have long been used for leiomyosarcoma with limited efficacy (19). Palbociclib has been shown to be effective for leiomyosarcoma, which may be due to a CDKNA2 deletion being found in 11%–32% of leiomyosarcoma patients who have a worse prognosis (22, 23). Clinical trials examining the efficacy of CDK4/6 inhibitor monotherapy in leiomyosarcoma have not been published.

A peritoneal-metastatic leiomyosarcoma PDOX model was established using SOI to implant a tumor fragment on the dome of the urinary bladder of nude mice (**Figure 1B**) (21). Palbociclib was administered to this recurrent leiomyosarcoma PDOX model for 21 days. Palbociclib showed significant efficacy on the PDOX growth, compared to the control group with a decreased number of cancer cells found in the treated tumor shown by histological analysis. The combination of gemcitabine and docetaxel was more effective (21). This report indicated moderate efficacy of palbociclib as a monotherapy in leiomyosarcoma. The status of CDKNA2 in this leiomyosarcoma PDOX tumor will be analyzed in a future study.

Dedifferentiated liposarcoma, which often occurs in the extremities and the retroperitoneal space, is a subtype of liposarcoma, with the lowest survival rate among liposarcomas (24). Amplification of CDK4 and murine double minute 2 (MDM2) is observed in 90% of dedifferentiated liposarcoma, suggesting the usefulness of palbociclib (25). Several clinical trials investigating the efficacy of CDK4/6 inhibitors for dedifferentiated liposarcoma have been performed or are ongoing (16). However, it should be noted that many of these clinical trials have analyzed dedifferentiated and well-differentiated liposarcomas together, which can have totally different clinical outcomes. When limited to dedifferentiated liposarcoma, interim analysis of a phase II study of abemaciclib, a CDK4/6 inhibitor, in patients with dedifferentiated liposarcoma demonstrated favorable outcomes (30.4 months in median progression-free survival) (26).

A retroperitoneal dedifferentiated liposarcoma PDOX model was established using SOI, by implanting the tumor into the retroperitoneum of nude mice by splitting the obliquus externus abdominis muscle (**Figure 1B**) (27). Palbociclib was administered to this liposarcoma PDOX model for 14 days. Palbociclib showed significant efficacy on PDOX growth compared to that of the control group. The tumor treated with palbociclib showed altered cancer cell shapes with an area of necrotic cells and fibrosis shown by histological analysis (27).

This report suggests that palbociclib is only moderately effective as monotherapy even in dedifferentiated liposarcoma that usually has CDK4 amplification.

## Combination treatment using palbociclib in soft-tissue sarcoma patient-derived orthotopic xenograft models

We tested combination treatment with palbociclib for increased efficacy on sarcoma. Recent studies have shown a synergy of CDK inhibitors with other chemotherapy drugs (16, 28). A phase II study of the combination of ribociclib, a CDK4/6 inhibitor, and everolimus, a mammalian target of rapamycin (mTOR) inhibitor, in patients with dedifferentiated liposarcoma and leiomyosarcoma is currently ongoing (ClinicalTrials.gov).

We have developed recombinant methioninase to restrict methionine in cancer cells (29). Methionine addiction is a fundamental and general hallmark of cancer and is termed the Hoffman effect (30). Methionine addiction appears to be caused by excess transmethylation reactions in cancer cells. Therefore, methionine restriction has been shown to be effective in many cancer types (29–32). Methionine-restricted cancer cells selectively arrest in late S/G2 of the cell cycle that may elicit a synergistic efficacy with palbociclib (32).

A methioninase and palbociclib combination was administered to the dedifferentiated liposarcoma PDOX model, described above, for 14 days. While monotherapy with palbociclib showed only moderate efficacy, similar to the study described above, the combination of methioninase and palbociclib regressed the PDOX tumor with extensive tumor necrosis. This report suggests that although palbociclib as a single agent is effective in treating drug-resistant soft-tissue sarcoma, the combination of palbociclib and other anticancer agents, including experimental drugs, may more effectively regress the tumors.

## Combination treatment using palbociclib in sarcoma patient-derived orthotopic xenograft models

Osteosarcoma is the most common malignant primary bone tumor (12). Resistance to traditional first-line chemotherapy drugs, such as doxorubicin and cisplatinum, leads to local and distance recurrence that is often fatal to patients, which are mostly adolescents and young adults (12). Osteosarcoma tends to have abnormal cell cycle control regulators, including CDKN2A and CDK4 (16). It has been reported that upregulated CDK4 expression in osteosarcoma patients correlates with the incidence of metastasis and poor prognosis, suggesting that palbociclib can be effective for osteosarcoma (33). However, prospective clinical trials investigating the efficacy of CDK4/6 inhibitors on osteosarcoma have not yet been performed (16).

**Figure 2:**
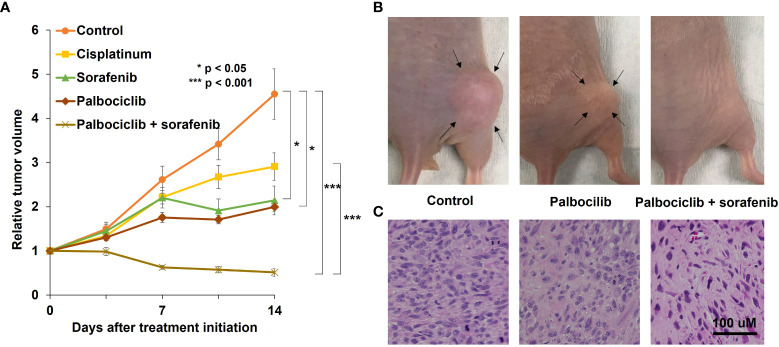
A representative osteosarcoma patient-derived orthotopic xenograft (PDOX) study identifying effective combination treatment with palbociclib. **(A)** The line graphs indicate the tumor volume at each time point after treatment start relative to the initial tumor volume for each group. *p < 0.05; ***p < 0.001. Error bar: ± standard error of the mean. **(B)** Representative photographs of the control, palbociclib-treated, or palbociclib–sorafenib combination-treated osteosarcoma PDOX models. Arrows indicate the tumor margins. **(C)** Hematoxylin and eosin-stained sections of control, palbociclib-treated, or palbociclib–sorafenib combination-treated tumors. Scale bar: 100 µm. Modified after Higuchi et al. (35).

The osteosarcoma PDOX model was established using SOI by implanting a tumor fragment into a space made by cutting the lateral condyle of the distal femur of nude mice (**Figure 1C**) (12). The patient tumor used for this osteosarcoma PDOX model was from a fresh biopsy sample of a pelvic osteosarcoma (34). Palbociclib either alone or combined with sorafenib was administered to the osteosarcoma PDOX model for 14 days (**Figure 2**) (35). Monotherapy with palbociclib significantly, but moderately, inhibited osteosarcoma PDOX growth and decreased cancer cell density (**Figure 2**). The combination of palbociclib and sorafenib significantly inhibited and regressed the osteosarcoma PDOX and extensively induced tumor necrosis with non-viable cells and degenerative changes in the stroma (**Figure 2**). Sorafenib is an oral multikinase inhibitor approved for the treatment of renal cell carcinoma, hepatocellular carcinoma, and thyroid cancer (36). The combination of sorafenib and palbociclib has been reported to have synergy against pancreatic carcinoma and hepatocellular carcinoma (35). The efficacy of the sorafenib–palbociclib combination to regress the osteosarcoma PDOX tumor indicates future clinical efficacy.

Another osteosarcoma PDOX was treated with palbociclib combined with everolimus, which is an mTOR inhibitor approved for breast and renal cell cancer (37). The patient tumor used for this osteosarcoma PDOX model was from a fresh surgical sample of femoral osteosarcoma (38). Monotherapy with palbociclib moderately inhibited the PDOX growth compared to that of the control (38). The combination of palbociclib and everolimus significantly inhibited tumor growth and induced cancer necrosis (38). This was the first report demonstrating the efficacy of the palbociclib–everolimus combination for osteosarcoma, suggesting promising clinical efficacy (38). CDK4/6 inhibition was reported to downregulate the downstream mediators of the mTOR pathway in several cancer types, predicting synergy of a CDK4/6 inhibitor combined with an mTOR inhibitor (39). The efficacy of this combination *in vitro* and *in vivo* was also shown in breast cancer, malignant pleural mesothelioma, and glioblastoma (39).

## Message to the reader

Sarcoma is a rare and heterogeneous group of cancers. Many molecular-targeted drugs have been developed for major cancers. However, prospective trials evaluating these drugs are limited in sarcoma due to its rarity and heterogeneity. Sarcomas have a poor prognosis when they are resistant to first-line chemotherapy. Therefore, discovering more effective treatment in sarcoma is mandatory.

The sarcoma PDOX model presents an opportunity to discover candidate unproven therapeutics for sarcoma, including the CDK4/6 inhibitor palbociclib. CDK4/6 inhibitors represent a potential breakthrough in cancer treatment. Although many sarcoma types have alterations in the CDK4/6 pathway, so far, no CDK4/6 inhibitor is approved for sarcoma treatment. We have reported six studies evaluating palbociclib as a candidate for sarcoma treatment using sarcoma PDOX models (**Table 1**). Palbociclib in combination with other agents effectively arrested the growth of all sarcoma PDOX tumors, showing that a CDK4/6 inhibitor is active against sarcoma. To regress the sarcoma PDOX tumors, palbociclib was used with other chemotherapy drugs, including an mTOR inhibitor, a multikinase inhibitor, and methioninase.

**Table 1:** Summary of efficacy of CDK4/6 inhibitors in the sarcoma PDOX studies.

Sarcoma type (Ref)	Presentation	SOI	Tested drugs (Standard chemotherapy and palbociclib)	Result
Ewing sarcoma (13)	Primary	Chest wall	Doxorubicin Linsitinib (not approved for sarcoma) **Palbociclib**	Total resistance Highly inhibited Arrested
Leiomyosarcoma (21)	Metastatic	Peritoneum	Doxorubicin Gemcitabine + docetaxel Pazopanib **Palbociclib**	Mildly inhibited Regressed Mildly inhibited Mildly inhibited
De-differentiated liposarcoma (27)	Recurrent	Retroperitoneum	Doxorubicin Pazopanib Gemcitabine + docetaxel Trabectedin Eribulin **Palbociclib**	Total resistance Mildly inhibited Mildly inhibited Mildly inhibited Regressed Mildly inhibited
De-differentiated liposarcoma (32)	Recurrent	Retroperitoneum	Doxorubicin rMETase (experimental) **Palbociclib** **Palbociclib** + methioninase	Total resistance Mildly inhibited Mildly inhibited Regressed
Osteosarcoma (35)	Primary	Femur	Cisplatinum Sorafenib (not approved for sarcoma) **Palbociclib** **Palbociclib** + sorafenib	Total resistance Highly inhibited Highly inhibited Regressed
Osteosarcoma (38)	Primary	Femur	Doxorubicin Everolimus (not approved for sarcoma) **Palbociclib** **Palbociclib** + everolimus	Total resistance Mildly inhibited Mildly inhibited Arrested

CDK, cyclin-dependent kinase; PDOX, patient-derived orthotopic xenograft; SOI, surgical orthotopic implantation.

CDK inhibitors are used preferentially in combination with other chemotherapy drugs that target dual genomic derangements and convert temporary cell cycle inhibition into permanent cell growth arrest or cell death (40). Although none of the nude mouse PDOX models used in the studies reviewed in the present report had significant toxicity such as significant weight loss or mouse death, combination chemotherapy in the clinic may have toxicity issues. Pharmacogenomic biomarkers are expected to identify effective drugs for sarcoma patients, thereby avoiding unnecessary drug toxicity (41). However, there are still no established biomarkers for each sarcoma subtype due to tremendous heterogeneity of this class of diseases. Further genetic and molecular biological analysis combined with drug–response studies using the PDOX model should contribute to the establishment of pharmacogenomic biomarkers in sarcoma patients.

Since the sarcoma PDOX model behaves similarly to the sarcoma in the patient, the results obtained here are directly relevant to clinical outcome (1). The present review demonstrated that a CDK inhibitor is active in the major types of sarcomas and that treatment using the CDK4/6 inhibitor palbociclib especially in combination with methionine restriction by methioninase or with a multikinase inhibitor is a promising strategy for sarcoma treatment in the clinic.

## Author contributions

Conception and design: TH and RMH. Acquisition, analysis, and interpretation of data: TH, KI, NY, KH, HK, SM, and MB. Writing, review, and revision of the article: TH, HT, and RMH. All authors contributed to the article and approved the submitted version.

## Funding

This work was supported in part by the Japan Society for the Promotion of Science (JSPS) KAKENHI Grant Number JP20K22802 and the Robert M. Hoffman Foundation for Cancer Research.

## Conflict of interest

Authors TH and RH are unpaid associates of AntiCancer, Inc.

The remaining authors declare that the research was conducted in the absence of any commercial or financial relationships that could be construed as a potential conflict of interest.

## Publisher’s note

All claims expressed in this article are solely those of the authors and do not necessarily represent those of their affiliated organizations, or those of the publisher, the editors and the reviewers. Any product that may be evaluated in this article, or claim that may be made by its manufacturer, is not guaranteed or endorsed by the publisher.
